# Mental Toughness and Individual Differences in Learning, Educational and Work Performance, Psychological Well-being, and Personality: A Systematic Review

**DOI:** 10.3389/fpsyg.2017.01345

**Published:** 2017-08-11

**Authors:** Ying Lin, Julian Mutz, Peter J. Clough, Kostas A. Papageorgiou

**Affiliations:** ^1^Department of Psychology, University of Southern California, Los Angeles CA, United States; ^2^Department of Epidemiology and Biostatistics, School of Public Health, Faculty of Medicine, Imperial College London London, United Kingdom; ^3^Manchester Metropolitan University Manchester, United Kingdom; ^4^Department of Psychology, Huddersfield University Huddersfield, United Kingdom; ^5^Queen’s University Belfast Belfast, United Kingdom; ^6^Department of Psychology, Tomsk State University Tomsk, Russia

**Keywords:** mental toughness, educational performance, psychological well-being, personality, genetics of mental toughness

## Abstract

Mental toughness (MT) is an umbrella term that entails positive psychological resources, which are crucial across a wide range of achievement contexts and in the domain of mental health. We systematically review empirical studies that explored the associations between the concept of MT and individual differences in learning, educational and work performance, psychological well-being, personality, and other psychological attributes. Studies that explored the genetic and environmental contributions to individual differences in MT are also reviewed. The findings suggest that MT is associated with various positive psychological traits, more efficient coping strategies and positive outcomes in education and mental health. Approximately 50% of the variation in MT can be accounted for by genetic factors. Furthermore, the associations between MT and psychological traits can be explained mainly by either common genetic or non-shared environmental factors. Taken together, our findings suggest a ‘mental toughness advantage’ with possible implications for developing interventions to facilitate achievement in a variety of settings.

## Introduction

Negative life events, crises, challenges, and stressful situations constitute a large aspect of human experience and are often unavoidable. For many individuals, the consequences of adversity negatively affect both physical and mental health and are frequently related to impairments in social, educational, and occupational functioning (e.g., Price et al., 2002; Springer et al., 2007; Scott et al., 2011). Since some individuals seem to do better than others in dealing with hardship, researchers, policy makers, and the public developed an interest in understanding the factors and processes that enable some individuals to persist when others give up.

Mental toughness (MT) has been studied as an important individual difference factor that allows individuals to deal effectively with challenges and to persist under pressure. MT has its highest profile in sport but its impact is now recognized in a wide range of other domains. It is an umbrella term that entails positive psychological resources, which are important across a range of achievement contexts (Clough et al., 2002; Crust and Clough, 2011; Gucciardi et al., 2015a). Moreover, it does not only reflect an effective coping mechanism as reaction to stressors (e.g., reappraising stressful situations as opportunities for self-development) but also allows individuals to proactively seek out opportunities for personal growth due to high levels of confidence in one’s abilities (St Clair-Thompson et al., 2015).

A number of MT models have been developed (e.g., Fourie and Potgieter, 2001; Clough et al., 2002; Jones et al., 2002; Golby and Sheard, 2006; Golby et al., 2007; Gucciardi et al., 2008; Coulter et al., 2010). For example, Clough et al. (2002) drew from hardiness theory to develop a multidimensional model of MT, whereas Gucciardi et al. (2015a) drew from theories of stress and personal resources to develop a unitary model of MT. While these models differ in several aspects, they also share a number of features. For instance, self-belief is at the core of most definitions (e.g., Clough et al., 2002; Bull et al., 2005; Thelwell et al., 2005; Gucciardi et al., 2008).

Moreover, an ever-increasing number of measurement scales have been developed. These include the Psychological Performance Inventory (Loehr, 1986), the Psychological Performance Inventory – A (Golby et al., 2007), the Mental Toughness Questionnaire 48 (MTQ48; Clough et al., 2002) and its short version the Mental Toughness Questionnaire 18 (MTQ18; Clough et al., 2002), the Mental, Emotional, and Bodily Toughness Inventory (MeBTough; Mack and Ragan, 2008), the Sport Mental Toughness Questionnaire (SMTQ; Sheard et al., 2009), the Cricket Mental Toughness Inventory (CMTI; Gucciardi and Gordon, 2009), the Australian Football Mental Toughness Inventory (Gucciardi et al., 2009a), the Mental Toughness Scale (Madrigal et al., 2013), the Mental Toughness Index (MTI; Gucciardi et al., 2015a), and the Military Training Mental Toughness Inventory (MTMTI; Arthur et al., 2015).

The many models and theories of MT share a core of concepts; this plethora of theories has led to the development of a number of questionnaires. Most of these have a limited validation history, which makes it very difficult to critique them. The MTQ48 is by far the most widely used and researched measure, based on the Clough et al.’s (2002) 4C’s model. Whilst there is still a debate about its appropriateness in some areas, it can be viewed as the ‘standard’ measure of MT to date (Clough et al., 2002). Drawing on the 3C’s model of hardiness (Kobasa, 1979), Clough et al. (2002) characterized MT as composite of four interrelated but independent subcomponents: (1) *control* (life and emotion): the tendency to feel and act as if one is influential and keep anxieties in check; (2) *commitment*: the tendency to be deeply involved in pursuing goals despite difficulties that arise; (3) *challenge*: the tendency to see potential threats as opportunities for self-development and to continue to strive in changing environments; and (4) *confidence* (in abilities and interpersonal): the belief that one is a truly worthwhile person in spite of setbacks, and the ability to push oneself forward in social settings.

Clough et al. (2002, p. 38) described mentally tough individuals “as tending to be sociable and outgoing as they are able to remain calm and relaxed, they are competitive in many situations and have lower anxiety levels than others. With a high sense of self-belief and an unshakable faith that they control their own destiny, these individuals can remain relatively unaffected by competition or adversity.”

There are similarities but also important differences between MT, hardiness, and resilience. While the 4C’s model of MT shares some conceptual foundation with hardiness, it clearly differs in its additional emphasis on confidence in one’s abilities and interpersonal relations. Hardiness is described by Kobasa (1979) as a personality disposition that provides resistance to stress. However, individuals who score high on MT are not only able to remain committed when confronting with stress, they are also confident about successfully completing the task and are assertive in social situations.

Mental toughness shares similarity with resilience in that both concepts promote positive adaptation in the face of adversity. Resilience is defined as “a phenomenon or process reflecting relative positive adaptation despite experience of significant adversity or trauma” (Luthar et al., 2006, p.742). MT is distinct from resilience in two important ways: first, resilience is a broad construct that encompasses a range of protective processes (e.g., biological and social factors), and is hence not directly measured but rather indirectly inferred (Luthar et al., 2006), whereas MT is a measurable as a specific set of traits. Second, the concept of resilience presupposes the existence of risk in the environment, but MT does not. MT not only relates to an individual’s reactions to risk and stress but also entails a proactive tendency to seek out challenges for personal growth (Gucciardi, 2017).

### Conceptual Debates

Many of the recent publications relating to MT have focused on its conceptual definition or the factor structure of its measures. Specifically, two main conceptual areas remain unresolved: (a) to what extent is it a trait (vs. a state), and (b) is it a unitary or multi-facet concept.

#### To What Extent Is Mental Toughness a Dispositional Trait

This discussion pertains to whether MT can best be conceptualized as a stable personality trait or as a mindset that varies highly across situations and over time. This is an important topic to investigate because MT is vital for high performance in competitive environments and stressful situations (Kaiseler et al., 2009). The notion that MT is adaptable would suggest that modifying the context in which individuals operate (e.g., the classroom environment) would allow individuals to perform to the best of their abilities. Furthermore, the notion that MT can be developed over time has useful implications for developing training programs aimed at increasing MT in order to boost performance and to improve general well-being. For example, training on aspects of MT such as goal-setting and self-reflection (Crust and Clough, 2011) may have substantial benefits in terms of encouraging attendance and enhancing academic performance (St Clair-Thompson et al., 2015).

Recent studies of MT that compare between-person (i.e., trait) versus within-person (i.e., state) variation as well as the role of genetic versus environmental factors provide insight into the extent to which MT is a trait. In a study conducted by Gucciardi et al. (2015a; Study 4), MT was measured once a week for 10 consecutive weeks. It was shown that 44% of the variance in MT was due to between-person variation and the rest was due to within-person variation. Evidence for environmental contributions also comes from behavioral genetic studies, which show that around half of the variance in MT is attributable to non-shared environmental factors (e.g., Horsburgh et al., 2009; Veselka et al., 2009). The fourth section “Genetics Research” provides more details about the role of genetic and environmental factors on individual variation in MT. Taken together, these studies support the idea that although there is considerable stability, MT is to some extent sensitive to environmental influences over time.

Clearly linked to this trait-versus-state debate is the possibility of developing MT. It has been suggested that MT can be developed through interventions that target elements of MT (Gucciardi et al., 2009a,b; Bell et al., 2013) and through positive youth experiences (Gould et al., 2011; Gucciardi and Jones, 2012). For example, Gucciardi et al. (2009a,b) evaluated the impact of two psychological skills training (PST) programs on athletes’ MT levels. One PST program focused on MT specifically, while the other focused on self-regulation, arousal regulation, mental rehearsal, attentional control, self-efficacy, and ideal performance states. Both interventions worked to increase subsequent ratings of MT. With regards to positive youth experiences, Jones and Parker (2013) showed that initiative experiences were the most important youth experiences in terms of their association with MT, and therefore may be particularly worth promoting. In addition, a study showed that psychological profiles in childhood predicted levels of MT in adolescence (Sadeghi Bahmani et al., 2016). Higher prosocial behavior, lower internalizing (e.g., feelings of sorrow, guilt, worry, and somatization) and externalizing problems (e.g., disruptive, disobedient, and harmful behavior) as well as more positive peer relationships rated by parents and teachers at age five predicted higher MT at age 14 among 77 adolescents. These lines of evidence suggest that MT is malleable to some extent and can be shaped by life experiences.

#### Dimensionality of Mental Toughness

Some researchers have argued for multidimensionality (e.g., Clough et al., 2002; Jones et al., 2002; Sheard et al., 2009; Coulter et al., 2010), whereas others have suggested that a unitary model is most appropriate (e.g., Gucciardi et al., 2015a). At the core of this debate lies an important discussion regarding the statistical models that have been used in different studies. It is clear that both approaches produce an acceptable model fit using statistical techniques such as confirmatory factor analysis (CFA), but that a unidimensional approach produces a better fit. However, this ‘better fit’ might come at a cost of reduced discrimination and, more importantly, a reduced focus for potential interventions. The reduction in degrees of freedom means fewer landing points when calculating an individual mean (fewer possible mean values for individual) and therefore less sensitivity.

This issue has prompted some authors to question the use of the CFA approach in model testing (e.g., Clough et al., 2012; Perry et al., 2015). Simplification of a measure to meet arbitrary statistical requirements, such as a certain model fit, may lead to a generic model and score. It is noted that this is a data-driven approach that leads to the arbitrary statistical requirements. If the overall score is made up of different elements of the concept that cannot be separated, any potential intervention must be generic and hence not targeted. With a multidimensional model one can identify the gap (e.g., low confidence) and aim to boost it through a more targeted intervention.

### The Current Review

The aim of the current review is (1) to provide a comprehensive summary and critical review of research on MT and (2) to identify limitations in the literature and provide an agenda for future studies on MT. We present findings from empirical studies that explored the degree to which individual differences in MT associate with individual variation in learning, educational and work performance, psychological well-being, personality and other psychological attributes, and genetics. Given that the concept of MT has extensively been reviewed in the context of sport and sport performance (Crust, 2007; Gerber, 2011; Chang et al., 2012), the present review focuses on studies that have implications beyond the sport arena. We do not intend to theoretically review the concept of MT and how it might relate to other concepts (interested readers may consult Crust, 2008).

In the authors’ view, MT has too often been restricted to sport and performance environments. We think that MT is an important concept that needs to be shown to be relevant to a wide range of applied and theoretical domains. The broad focus on other areas (e.g., genetics), is important as it again demonstrates its relevance and integrity across a range of psychological domains. An abundance of sport-specific empirical studies and reviews (e.g., Gucciardi et al., 2009c) have been carried out to link MT with successful sport outcomes. The importance of MT to sport settings is well established, but less is known about MT’s association with psychological traits and performance in other (than sports) contexts. Most of the literature on these topics has emerged in the past decade. It is precisely due to this contrast of knowledge on the link between MT in sport vs. non-sport domains that we decided to narrow the scope of our review to focus on MT studies outside sport settings.

## Materials and Methods

### Literature Search

A systematic literature search was conducted using the PsycINFO, Embase, MEDLINE, Scopus, and Web of Science databases. We searched for articles published from the first date available to October 2016 using the key words “mental toughness” OR “mentally tough” without any limits. Once all relevant studies were retrieved, their titles and abstracts were screened and selected by means of prespecified inclusion/exclusion criteria (outlined below). In cases where the title and abstract provided insufficient information to determine whether a study could be included, the full-text version of the article was screened. Additionally, we hand-searched reference lists of published articles on MT for further studies. One author (JM) conducted the literature search and screened relevant articles against the inclusion/exclusion criteria. The final list of included studies was approved by all authors.

### Inclusion and Exclusion Criteria

#### Inclusion Criteria

Included studies were required to meet the selection criteria. Specifically, we included (1) quantitative studies that were published in a peer-reviewed scientific journal, (2) articles that were written in English, (3) studies that associated MT with non-sport related measures; studies that assessed MT in athletic populations were included under the condition that athletes’ MT was associated with athletes’ cognitive and behavioral traits (and not their performance in sport *per se*), and (4) studies on the genetics of MT. These are included because they also report phenotypic associations between MT and psychological/personality traits; and because they provide evidence on the degree to which variation in MT is influenced by environmental factors.

#### Exclusion Criteria

We excluded editorials, reviews, qualitative studies, and research that examined MT solely in relation to sport outcomes (e.g., swimming performance). There are two main reasons why qualitative studies are not included in this work: Firstly, nearly all the qualitative studies have focused on defining and conceptualizing MT. This is beyond the scope of this paper. In addition, the nature of qualitative research means it is often designed to explain a particular example and has less focus on replicability and comparability. We also excluded studies that examined the relationship between MT and demographic variables or which examined the MT profile of individuals at different levels of competition.

### Data Extraction

For each paper that met the criteria for inclusion in our review, we extracted the following data: authors, year of publication, sample size and population, measure of MT, related attributes that were assessed (and the measures being used) as well as details about the primary findings.

## Results

**FIGURE 1:**
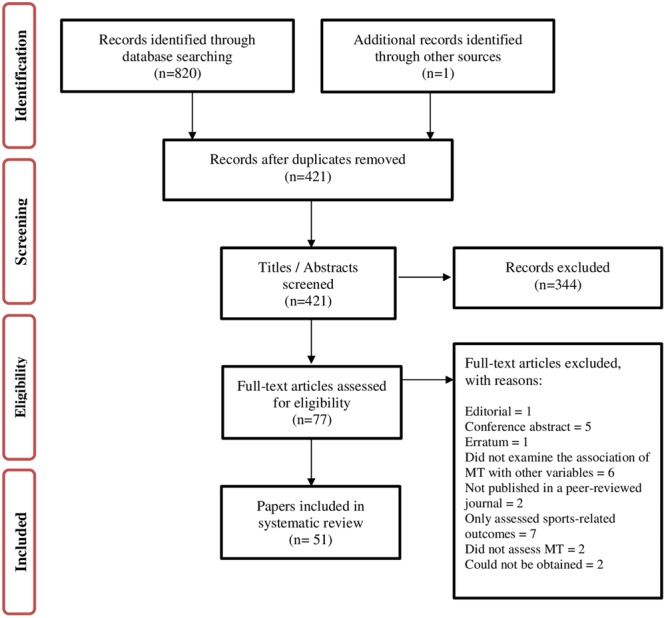
Preferred Reporting Items for Systematic Reviews and Meta-Analyses (PRISMA) flow diagram of literature search.

A Preferred Reporting Items for Systematic Reviews and Meta-Analyses (PRISMA; Moher et al., 2009) flowchart is presented in **Figure 1**. Fifty-one papers (describing 56 studies) were included in our review. Supplementary Table S1 provides a quick look summary of all studies that examined the relationship between individual differences in MT and individual variation in learning, educational and work performance (section 1), psychological well-being (section 2), personality and other psychological attributes (section 3), and genetics studies of MT (section 4).

### Section 1: Learning and Educational and Work Performance

In this section, we review studies that examined the degree to which individual differences in MT associate with education and learning outcomes. Moreover, we discuss the usefulness of MT as a predictor of performance in a range of other domains.

#### Education and Learning

To identify the cognitive mechanisms underlying MT, Dewhurst et al. (2012) examined the relationship between MT and performance on the directed forgetting paradigm (Bjork, 1970). Participants with different levels of MT studied two lists of words (List 1 and 2) and were told to forget List 1. They were then given a surprise memory test that included items from both lists. On average, mentally tough individuals performed better at recalling items form List 2 when they had received instructions to forget List 1. The commitment dimension of MT (i.e., the ability to involve oneself and concentrate on the current task) correlated most strongly with performance on the directed forgetting paradigm. As such, the researchers suggested that cognitive inhibition, the ability to ignore irrelevant stimuli and to focus on the requirements of a given task, might be one of the mechanisms underpinning MT. A more recent study by Delaney et al. (2015) replicated Dewhurst et al.’s (2012) findings and showed that mentally tough undergraduates performed better at the directed forgetting paradigm. They also demonstrated that individuals who scored high on MT and the Big Five trait of conscientiousness were more likely to invest great effort to switch encoding strategies and to forget irrelevant information, likely contributing to better memory performance (Delaney et al., 2015).

It is, however, important to note that the explanatory power of MT in cognition may differ depending on whether MT is conceptualized as a general or a domain-specific trait. For example, Hardy J.H. et al. (2014) asked university students to complete questionnaires that assessed trait-based (the 18-item MTQ) and domain-specific (e.g., “I can handle any game put in front of me”) MT. Subsequently, the students underwent a number of practice trials and performed a computer game that involved a strong component of complex task learning. Domain-specific MT predicted post-practice self-efficacy and performance on the learning task. In contrast, trait-based MT did not relate to any of the learning and performance criteria in the study. Hence, the researchers raised concerns about the predictive value of MT as a general (as opposed to domain-based) trait in the context of learning outcomes (Hardy J.H. et al., 2014).

These studies demonstrate that MT has potential value for application in educational settings. Crust et al. (2014) first investigated the predictive value of MT in the sport-related academic domain. They used the MTQ48 to predict academic grades in a sample of 161 first year sport students and showed that those students who scored high on MT achieved higher grades compared to those who had lower levels of MT. Moreover, students who achieved passing grades reported higher MT than those students who failed the academic year. Using the MTI, Gucciardi et al. (2015a; Study 4) examined the associations between MT and self-reported psychological health, thriving, and goal progress both in academic and social domains over 10 weeks in 203 undergraduates. Both within-person and between-person differences in MT were used as predictors in a multilevel structural equation model. At both levels of analysis, MT emerged as a predictor of more positive and fewer negative emotional states, higher thriving as well as more academic and social goal progress.

St Clair-Thompson et al. (2015; Study 1) explored the degree to which individual differences in MT associate with individual variation in secondary school performance. In a sample of 159 adolescents, they observed positive correlations between total MT and academic attainment/attendance. Furthermore, the challenge, commitment, and control subscales (overall control and life control) of the MTQ48 yielded particularly high correlations with both academic variables. A conceivable explanation of these findings is that individuals scoring high on MT perceive themselves as being influential in creating their own future: they find it easier to cope with school-related stressors, and they are able to effectively deal with threats to their attendance such as illness or dysfunctional family environments. Positive correlations of the emotion control and confidence in own abilities subscales with classroom attendance were also observed (St Clair-Thompson et al., 2015, Study 1). In a second study with 259 adolescents, they observed that total MT, commitment, control, life control, and interpersonal confidence negatively correlated with counterproductive classroom behavior (St Clair-Thompson et al., 2015, Study 2). This finding is in line with previous theoretical accounts, which suggest that students who are highly committed to a particular task tend to be more focused, diligent, and engaged with their learning environment (Clough and Strycharczyk, 2012). In a third study by the same group, St Clair-Thompson et al. (2015; Study 3) showed that in a sample of 93 adolescents, total MT and the scales of confidence in abilities, interpersonal confidence, and overall levels of confidence were significantly associated with peer relationships at school, as measured by social inclusion. Interestingly, individuals were more likely to interact with those who scored high on interpersonal confidence (possibly reflecting self-esteem) and to work with those who scored high on confidence in abilities (possibly reflecting self-efficacy).

In a more recent study, St Clair-Thompson et al. (2017; Study 1) showed that in a sample of 105 pupils all subscales of the MTQ48 correlated positively with explicit self-esteem, while MT was associated with fewer concerns about moving to a new school. Importantly, the confidence in own abilities subscale was predictive of fewer school concerns over and above the variance that could be explained by individual differences in self-esteem. St Clair-Thompson et al. (2017; Study 2) also showed that in undergraduate students all subcomponents of MT were positively associated with academic, social, and personal-emotional adjustment as well as institutional attachment. More specifically, commitment and life control significantly predicted academic adjustment, life control and interpersonal confidence predicted social adjustment, and commitment, emotional control, and confidence in one’s abilities predicted personal-emotional adaptation. Only life control was a statistically significant predictor of institutional attachment. Both of these studies suggest that MT is a relevant concept when it comes to educational transitions, with potential implications for educational practice.

Taken together, these findings suggest that MT is a valuable concept in educational settings and other learning environments. Studying MT in relation to cognition and educational performance can shed light on the psychological resources that contribute to individual variation in these domains. It might also facilitate the development of effective interventions aimed at improving school and academic performance (St Clair-Thompson et al., 2015).

#### Work Place

Increasing attention has been directed to the relationship between MT and performance in domains other than sport or education. The association between MT and work performance was first documented by Marchant et al. (2009) who showed that higher levels of MT were associated with more senior managerial positions in a sample of 522 individuals working in United Kingdom-based organizations. However, due to the cross-sectional nature of the study, it was unclear whether MT contributed to career achievement or whether holding more senior managerial positions helped develop higher levels of MT. In addition, Gucciardi et al. (2015a; Study 3) showed that scores on the MTI were directly related to higher levels of supervisor-rated work performance in a sample of 497 employees. Perceived stress was a statistically significant mediator in this relationship.

#### Military

Mental toughness may also be informative in military settings: Godlewski and Kline (2012) found that in a sample of 459 Canadian Forces recruits, higher levels of MT were associated with stronger commitment and better transitions, which in turn led to lower turnover intentions and behavior. In a study by Gucciardi et al. (2015a; Study 5), military candidates’ levels of MT were measured using the MTI, a scale that captures MT as a unidimensional construct. Their results showed that scores on the MTI predicted success on a military selection test over and above hardiness and self-efficacy in 115 male candidates. Because hardiness (control, commitment, challenge) in combination with self-efficacy may be similar to the four dimensions of the 4C’s model of MT, the authors suggested that the unidimensional conceptualization of MT predicted military performance better than the 4C’s model of MT would. Similarly, Arthur et al. (2015) developed a military training MT inventory, and MT scores derived from the inventory successfully predicted military course performance among 134 paratroop recruits over and above individual fitness. Given the relationships between MT, adjustment, turnover, and performance, general and domain-specific MT questionnaires might be useful screening tools for recruitment within a military context.

### Section 2: Psychological Well-being

Not only does MT play a role in educational and work settings, it is a concept of relevance to positive mental health outcomes and in enabling high performance in challenging situations. This section presents studies that have explored MT in relation to mental health and affective traits.

#### Mental Health

In a longitudinal study, Gerber et al. (2013a) explored the relationships between MT, perceived stress, depressive symptoms, and life satisfaction. Levels of perceived stress were assessed to provide an estimate of adverse life experiences; depressive symptoms and life satisfaction were assessed to estimate overall levels of adjustment (representing maladaptive and adaptive emotional development, respectively). In a sample of 865 students at vocational schools, both perceived stress and depressive symptoms correlated negatively with the scores on the MTQ18. Moreover, MT was positively associated with life satisfaction. The researchers also found that well-adjusted individuals (low levels of stress, few depressive symptoms, and high life satisfaction) scored high on MT, whereas maladjusted individuals (high levels of stress, depressive symptoms, and little life satisfaction) tended to have lower levels of MT. Resilient (moderate levels of stress at baseline, decreased depressive symptoms and increased life satisfaction at follow-up) and deteriorated (increasing levels of stress, increasing depressive symptoms, and decreasing life satisfaction) individuals did not differ at baseline but showed an increase/decline of MT over time (resilient and deteriorating individuals, respectively).

In line with these findings, Gerber et al. (2013b) showed that MT was associated with lower perceived stress and fewer depressive symptoms in a sample of 284 high school students and in a sample of 140 undergraduate students. They also showed that MT moderated the relationship between high stress and depressive symptoms. More specifically, high levels of MT were associated with lower depressive symptoms when stress levels were high. Since high levels of stress increase the risk for maladjustment and psychopathology (Grant et al., 2006), the influence of MT in promoting positive adaptation is of practical relevance. The authors suggested that training MT might be particularly relevant for those individuals who are typically difficult to be reached with more traditional health interventions (Gerber et al., 2013b).

Gucciardi and Jones (2012) showed small to moderate negative correlations between MT and stress, anxiety, and depression in a sample of 226 cricketers. This finding was replicated by Jin and Wang (2016) in a sample of 217 international students: higher levels of MT were associated with lower levels of stress, anxiety, and depression. The latter study also showed that MT was associated with better life satisfaction as well as less attachment anxiety and avoidance. A statistical mediation model that examined whether individual differences in MT mediate the relationship between adult attachment and psychological distress was only partially supported: MT mediated the relationship between attachment anxiety, but not attachment avoidance, and psychological distress and life satisfaction. The authors suggested that targeting MT rather than attachment styles to improve well-being might be more fruitful.

To further elucidate the relationship between MT and depressive symptoms, Mutz et al. (2017) showed that MT correlated negatively with the habitual use of expressive suppression (i.e., inhibiting emotion-expressive behavior) in a sample of 364 adults. Furthermore, they observed a positive correlation with the habitual use of cognitive reappraisal (i.e., reinterpreting the subjective meaning of emotion-eliciting stimuli to alter the emotional response). Individual differences in cognitive reappraisal and MT were negatively associated with depressive symptoms, whereas expressive suppression showed a positive correlation with depressive symptoms. A statistical mediation model provided tentative support for the hypothesis that the relationship between MT and depressive symptoms is mediated by individual differences in expressive suppression. However, no evidence could be obtained that individual differences in cognitive reappraisal mediated the association between MT and depressive symptoms (Mutz et al., 2017).

Psychological well-being is critical for achievement and for desirable life outcomes in domains including, but not limited to, work (Daniels and Harris, 2000), education (Chow, 2007), and interpersonal relationships (Pickett-Schenk et al., 2006). On the flip side, mental health problems are associated with poor academic performance, attrition, less days devoted to study, suicidal thoughts, and disordered eating (Duane et al., 2003; Kugu et al., 2006). In a sample of 168 undergraduate students, all components of the MTQ48 were found to be moderate to strong predictors of greater psychological well-being, which encompassed six distinct factors: (1) self-acceptance, (2) personal growth, (3) purpose in life, (4) positive relations with others, (5) environmental mastery, and (6) autonomy (Stamp et al., 2015).

#### Sleep Quality

Sleep quality appears to predict important psychological outcomes throughout development, including cognitive performance (Bub et al., 2011) and psychosocial symptoms of depression and anxiety (Gregory et al., 2005; El-Sheikh, 2011). Indeed, sleep patterns in childhood tend to have an ongoing impact on well-being and psychological functioning throughout development (Brand et al., 2015a). Given the relationship between MT and psychological functioning, such as less perceived stress, sleep quality might be improved in mentally tough individuals.

Several studies have explored the relationships between MT, sleep, and psychological functioning using samples of pre-schoolers and adolescents. The first study investigated the association between MT and objectively assessed sleep in a sample of 92 adolescents (Brand et al., 2014a). Individuals who scored high on MT displayed higher sleep efficiency, fewer awakenings after sleep onset as well as more deep sleep and rapid eye movement sleep. Furthermore, they reported lower daytime sleepiness and fewer sleep complaints, and their sleep electroencephalograph (sEEG) signal was indicative of better objective sleep quality. There was no evidence for a relationship between MT, sleep duration, and sleep onset latency. Similar results were obtained in a sample of 284 adolescents, except that the correlation between greater MT and shorter sleep onset latency was statistically significant. In addition, mentally tough individuals showed fewer depressive symptoms and less perceived stress (Brand et al., 2014b). Since both depression and stress constitute main reasons for sleep complaints (Riemann et al., 2010), the authors suggested that fewer psychopathological symptoms might explain the association between MT and sleep quality (Brand et al., 2014b).

A longitudinal study conducted by Brand et al. (2015a) investigated whether sleep patterns in childhood would predict MT and psychological functioning at a later stage of development. The study involved a sample of 37 adolescents, whose sleep quality had been assessed as pre-schoolers 9 years ago through sEEG and self-report. The results indicated that sleep quality at the age of five was related to a range of psychological functions at the age of 14, despite showing no association with current sleep patterns. More specifically, normal and good sleepers at the age of five demonstrated more favorable scores of MT, perceived stress, mood, self-perception, and school marks at the age of 14, relative to poor sleepers. However, it is worth mentioning that psychological functioning and MT was not assessed at the age of five, and the possibility of a bidirectional relationship between MT and sleep quality cannot be dismissed. More recently, Brand et al. (2016a) showed in a large sample of 1475 adolescents that greater MT was associated with fewer sleep disturbances and greater quality of life, which is in line with previous studies.

#### Stress Coping

Prolonged periods of high stress levels contribute to the development of mental health problems (Rabkin and Struening, 1976) and may impair cognitive functioning (Arnsten, 2009). Stress is often provoked by events that threaten one’s sense of adaptive adequacy because they exceed perceived capacities to meet the demands of the situation (Sherman and Cohen, 2006). Hence, the ability to cope well with stress is an important determinant of performance success under pressure situations (Kaiseler et al., 2009; Nicholls et al., 2009).

Recent works have explored relevant cognitive attributes in an attempt to explain why mentally tough individuals seem to cope more effectively and tend to perform better under pressure. Kaiseler et al. (2009) highlighted the association between MT and problem-focused coping (i.e., strategies used to decrease distress by reducing or eliminating the stressor). Mentally tough athletes reported more problem-focused coping strategies, while also using fewer emotion-focused and avoidance strategies in response to a self-selected stressor. At the same time, MT and problem-focused coping strategies were associated with greater self-ratings of coping effectiveness. Indeed, Nicholls et al. (2011) showed that MT was positively associated with both global coping self-efficacy and coping effectiveness in a sample of 206 athletes.

In line with these findings, MT has been linked to learned resourcefulness, a repertoire of acquired abilities that enable appropriate problem-solving, coping, emotional control, and behavioral responses (Cowden et al., 2014). Cowden et al. (2016) showed that both MT and resilience were associated with less stress in a sample of tennis players. This study is important in that it highlights conceptual parallels between the two concepts. Although the studies above focus on athletic populations, problem-focused coping has been demonstrated to be effective in mitigating negative impacts of stressors across diverse environments, including the workplace and at home (e.g., Parkes, 1990; Stoneman and Gavidia-Payne, 2006). It is possible that the relationship between MT and problem-focused coping is transferable to non-athletic populations and hence might have broad implications across diverse contexts.

Evidence that links MT with stress coping in domains other than sport comes from Gerber et al. (2015a) who showed that in vocational students, MT was not only associated with less stress but also fewer indices of burnout, including physical fatigue, cognitive weariness, and emotional exhaustion. In addition, there is mixed evidence that MT contributes to stress reduction by increasing exercise participation. Some studies have shown that mentally tough individuals were more likely to be involved in physical activity (Gerber et al., 2012) and were more likely to turn intentions to exercise into actual exercise behaviors (Hannan et al., 2015). While chronic exposure to stress may gradually lead to burnout (Riolli and Savicki, 2003), MT reduced burnout symptoms indirectly by promoting physical activity in 56 vocational students (Gerber et al., 2015b). Similarly, a large-scale study with 1,361 adolescents showed that levels of MT varied as a function of group differences in levels of physical activity. Compared to the low physical activity group, individuals in the high physical activity group had higher levels of MT (Brand et al., 2016b). On the other hand, Sabouri et al. (2016) showed that MT was associated with vigorous physical activity, but not moderate physical activity or walking, in a sample of 341 adults. However, a recent investigation by Brand et al. (2016a) did not find evidence of a positive relationship between MT and self-reported levels of physical activity in a large sample of 1,475 adolescents, raising doubts about the role of physical activity and exercise in mediating the relationship between MT and stress reduction. Potential confounders such as the amount and the type of exercise may contribute to the presence of mixed results. A closer look at the underlying mechanisms linking physical activity and exercise to MT may provide better insights into the circumstances under which physical activity and exercise contribute to greater MT and vice versa.

Few other mechanisms have been proposed to explain the relationship between MT and effective coping under pressure, including sensitivity to punishment, emotional intelligence, and low affect intensity. One study found that cricketers who were rated as mentally tough by their coaches tended to be more sensitive to punishment cues but not to reward cues (Hardy L. et al., 2014). Punishment sensitivity may predispose individuals to notice threats in their environment at an early stage and therefore provide them with more opportunities to initiate an effective response to these threats. Furthermore, mentally tough athletes were found to be more emotionally intelligent (Nicholls et al., 2015). It has been suggested that MT’s association with emotional intelligence may play a protective role against stress by reducing anxiety. With regards to affect intensity, it has been hypothesized that mentally tough individuals might generally be less affected by emotion-provoking stimuli (Crust, 2009) or have lowered levels of trait anxiety (Cowden et al., 2014). However, Crust (2009) found no significant association between affect intensity and MT in a sample of sport performers. Participants with varying levels of MT did not characteristically experience less intense emotions. Research also failed to demonstrate a link between MT and trait anxiety (Cowden et al., 2014). It seems that MT does not affect one’s tendency to experience a situation as stressful, but rather provides important coping resources in the face of adversity.

Taken together, these results suggest that efficient emotional control and the prioritization of problem-focused coping strategies over avoidance coping strategies might be important aspects of MT that are responsible for effective stress coping. Furthermore, MT is not only an important concept in performance contexts but also a vital resource relevant to mental health, positive psychological functioning, and general well-being.

### Section 3: Personality and Other Psychological Attributes

Mental toughness associates with several personality traits and other psychological attributes that are established predictors of performance across diverse settings. Examining the relationships between MT, personality, and other psychological attributes might contribute to our understanding of how MT links with achievement-related outcomes. This section reviews studies that explored the association between MT, personality traits, and any other psychological attributes that have not been reviewed earlier.

Mental toughness is likely to predict favorable performance outcomes in ways that promote motivation, self-efficacy, and striving across situations. Gucciardi (2010), Gucciardi and Jones (2012), and Gucciardi et al. (2015b) conducted several studies examining MT in relation to aspects of motivation in youth athlete samples. Australian football players with high levels of MT endorsed achievement goals more frequently and were highly motivated to play well compared to those with moderate levels of MT (Gucciardi, 2010). In another study, Gucciardi et al. (2015b) investigated the relationships between MT, inspiration, and passion in a sample of 347 teenage tennis players. Specifically, harmonious and obsessive passion was examined. While the former emphasizes autonomous internalization of an activity into one’s identity due to satisfaction derived from the activity *per se*, the latter involves internalization of an activity into one’s identity as a result of experiencing pressure. Higher levels of MT behaviors, rated by the players’ parents, were positively related to the players’ self-reports of harmonious passion and frequency of inspiration; and negatively related to their self-reports of obsessive passion and fear of failure (Gucciardi et al., 2015b).

The positive associations between MT and aspects of motivation have important affective consequences. Gucciardi and Jones (2012) showed that mentally tough cricketers not only possessed greater developmental assets, which are indicators of thriving, but also reported lower frequencies of negative emotional states compared to cricketers with lower levels of MT. Similarly, Schaefer et al. (2016) showed that golfers who reported higher levels of motivation had lower levels of competition anxiety, an association that is mediated by MT. These studies suggest that MT and motivation may work hand in hand to contribute to greater emotional stability and positive outcomes in demanding situations.

Meggs et al. (2014) indicated that some important characteristics of mentally tough individuals, including motivation and emotional resilience, stemmed from differences in evaluative self-organization (i.e., the organization of positive and negative self-beliefs). In their study, 105 athletes at a range of performance levels completed the SMTQ and a self-descriptive attribution task (Showers, 2002). Participants with higher levels of total MT had more positive than negative self-concepts. More specifically, self-concept was associated positively with greater levels of control and confidence. The notion that mentally tough individuals tend to perceive themselves and situations positively provides an account for why they tend to continue striving despite facing difficult situations.

Another line of research focused on flow as an explanation for MT’s association with high levels of performance (Crust and Swann, 2013). Flow is an intrinsically motivating experience in which one is fully immersed in what one is doing. Research has shown that flow experiences associate with high levels of motivation and performance in diverse performance domains, including sport and the workplace (Jackson and Eklund, 2002; Martin and Jackson, 2008). In their study, 135 athletes who scored high on the MTQ48 reported more frequent experiences of flow (Crust and Swann, 2013). In line with these findings, Madrigal et al. (2013) showed that 271 athletes with higher levels of MT, measured by the MTS, exhibited greater self-esteem, self-efficacy, competitiveness, goal orientation, and flow. All these factors may be relevant in elucidating the complex relationship between MT and high performance in competitive environments. It is worth noting that despite high levels of confidence in their abilities, mentally tough individuals do not seem to set unrealistically high and rigid performance standards. A study that looked at the relationship between perfectionism and MT showed that higher levels of MT were associated with lower levels of concerns about mistakes, doubts about actions, and excessive personal standards in a sample of 46 adult students (Brand et al., 2015b).

A recent study applied Basic Psychological Needs Theory (BPNT) to further explore the underlying mechanism between MT and positive psychological functioning (Mahoney et al., 2014). BPNT proposes that optimal functioning depends on the satisfaction of three fundamental psychological needs: autonomy, competence, and relatedness. Mahoney et al. (2014) proposed that an autonomy-supportive coaching environment is an indirect antecedent of MT by means of psychological needs satisfaction, which in turn, is related to adaptive outcomes including positive affect and high levels of performance. To test their hypotheses they collected self-report data on the MTI, the Basic Needs Satisfaction in Sport Scale (Ng et al., 2011), and race time measures from 221 cross-country runners. Psychological needs satisfaction enhanced perceptions of self-value, efficaciousness, and self-control, which may have contributed to the investment of greater effort despite facing challenges. This mechanism embedding MT may carry practical implications for changing environments to enhance MT and to produce desirable performance outcomes. It should be noted that while this model attempted to explore antecedents and consequences of MT, it was based on cross-sectional data. Therefore, it remains unclear whether MT is contingent on psychological needs satisfaction or whether mentally tough individuals are more capable of achieving satisfactory psychological states. Longitudinal or experimental research is needed to determine causality or potential reciprocity of these relationships.

Nicholls et al. (2008) explored the associations between MT, optimism, and coping in a sample of 677 athletes. Using the MTQ48, they found that MT was positively associated with optimism, and negatively associated with pessimism. Given that optimism has been associated with sustained efforts to achieve goals (e.g., Solberg Nes et al., 2005), the authors suggested that the interaction between MT and optimism might contribute to high achievement (Nicholls et al., 2008).

However, mentally tough individuals are also likely to demonstrate socially malevolent characteristics in order to achieve their goals. A recent study explored the association between MT and the Dark Triad personality traits of Machiavellianism, narcissism, and psychopathy (Sabouri et al., 2016). Individuals who score high on the Dark Triad traits tend to be “callous, selfish, and malevolent in their interpersonal dealings” (Paulhus and Williams, 2002, p. 100). Sabouri et al. (2016) showed that higher MT scores were associated positively with all facets of the Dark Triads among 341 Iranian athletes. It has been suggested that mentally tough individuals who are committed to their goals may be more likely to use exploitative social strategies that focus on self-interest and motivate striving.

In addition to research focusing on predictors of performance, Crust and Keegan (2010) explored the relationship between MT and risk-taking attitudes among 105 athletes. Overall MT, as measured by the MTQ48, was positively correlated with attitudes toward physical but not psychological risks. More specifically, individuals who scored high on the subscales of challenge and confidence in their abilities were more willing to take on physical risks, while individuals who had greater interpersonal confidence tended to take more psychological risks. However, the study measured attitudes toward risks rather than risk-taking behavior; as such, mentally tough individuals do not necessarily take more behavioral risk in decision-making. Behavioral measures of actual risk-taking would help solidify the findings.

In summary, MT associates with several established predictors of performance such as positive self-concepts, self-esteem, and self-efficacy. These correlates foster positive adaptation and striving in performance environments and may form part of the indirect paths that link MT with achievement-related outcomes. An implication of these finding is that positive psychological and behavioral outcomes may be cultivated by promoting MT. As such, it is essential to further explore the factors that contribute to individual variation in MT in the general population. The next section reviews behavioral genetic studies that aim to explore the degree to which individual differences in MT can be accounted for by genetic and environmental factors. The only molecular genetic study that investigated whether specific genes contribute to variation in MT is also reviewed.

### Section 4: Genetics Research

#### Behavioral Genetics Studies

Four behavioral genetics studies have been conducted to explore the degree to which individual variation in MT can be accounted for by genetic and environmental factors. All four studies have used the twin design, which is a major method in behavioral genetics research commonly used to disentangle genetic and environmental sources of resemblance between relatives (twins). If genetic factors are important for a trait, monozygotic (MZ) twins (genetically identical pairs of individuals) must be more similar (on the trait of interest) than fraternal (dizygotic, or DZ) twins, who are only 50% genetically similar (Plomin et al., 2013). The statistic that estimates the genetic effect size — the extent to which genetics contribute to a trait — is called heritability. Heritability is the proportion of phenotypic variance that can be accounted for by genetic differences among individuals (Plomin et al., 2013).

The first behavioral genetic investigation of MT aimed at exploring the relationship between MT and the Big Five personality traits at the phenotypic, genetic, and environmental levels in a sample of 219 adult twin pairs (Horsburgh et al., 2009). The heritability estimate for overall MT was 0.52; the remaining 48% of the variation in MT was accounted for by non-shared environmental factors; that is, environmental influences that make children growing up in the same family different, rather than similar. The heritability estimates for the subscales of the MT ranged from 0.36 (for commitment) to 0.56 (for emotional control; Horsburgh et al., 2009). The authors concluded that the scales of commitment and control can be easier to train given that variation in those components is explained mainly by environmental rather that genetic factors (Horsburgh et al., 2009).

Horsburgh et al. (2009) also found a negative phenotypic correlation between all MT subscales and neuroticism. Positive phenotypic correlations were found between MT and extraversion, openness to experience, agreeableness, and conscientiousness. The phenotypic correlations were attributable to genetic factors (suggesting that common genetic factors influence both traits) and, to a lesser extent, to correlated non-shared environmental factors. Genetic correlations between the MT subscales and the Big Five traits were moderately strong, varying from 0.23 (commitment and openness to experience) to 0.91 (overall control scale and neuroticism; Horsburgh et al., 2009).

Veselka et al. (2009) explored whether MT and trait emotional intelligence can be integrated into a general factor of personality (GFP). Evidence for the existence of a GFP was obtained representing high levels of MT, extraversion, and conscientiousness as well as low levels of neuroticism (Veselka et al., 2009). Genetic (53%) and non-shared environmental factors (47%) explained all the variance in the GFP.

Another study investigated the relationships between MT and humor styles (Veselka et al., 2010). MT was assessed using the MTQ48, while humor styles were assessed using the Humor Styles Questionnaire (Martin et al., 2003). The HSQ assesses individual differences in four humor styles, namely, affiliative, self-enhancing, aggressive, and self-defeating humor (Martin et al., 2003). Overall MT showed a positive association with affiliative and self-enhancing humor styles, and weak negative associations with aggressive and self-defeating humor. Behavioral genetic analyses revealed that both common genetic and environmental factors influence individual differences in MT and humor styles. The authors concluded that whilst MT may influence the humor style that one uses, it is also possible that the propensity to engage in different humor styles may allow for the development of greater or reduced MT (Veselka et al., 2010).

The most recent behavioral genetic study on MT explored the phenotypic association between the Dark Triad traits of psychopathy, Machiavellianism, and narcissism with MT in a sample of 210 adult same-sex twin pairs. They also explored the etiological factors underlying the correlations between these traits. The purpose of this investigation was to shed light on some of the factors that contribute to the success of individuals exhibiting the Dark Triad traits in workplace and social settings (Onley et al., 2013).

At the phenotypic level, all MT subscales were associated positively with narcissism and negatively with psychopathy (with the exception of the association between the challenge subscale of MT and psychopathy that was statistically not significant). Machiavellianism was positively associated with commitment and control and negatively associated with challenge and confidence. Behavioral genetic analyses indicated that the correlations between narcissism and MT (total and subscales) were attributable mainly to common non-shared environmental factors (15–23%). Correlations between Machiavellianism and MT (total and subscales) were influenced by both common genetic and non-shared environmental factors (34–49% and 16–26%, respectively). The correlations between psychopathy and the MT subscales were attributable entirely to correlated genetic factors (26–38%; Onley et al., 2013). The authors concluded that exploring the nature of the Dark Triad and its correlation with prosocial traits, such as MT, can shed light on both the adaptive and maladaptive qualities associated with the Dark Triad. It can also facilitate our understanding of the etiological factors (genetic and environmental) that explain how these traits interact to produce variation in performance in a variety of settings (Onley et al., 2013).

#### Molecular Genetics

There is only one preliminary molecular genetics study that has explored the association between genetic variants in the serotonin transporter (5-HTT) gene and a range of positive psychological attributes, including MT, in 31 national-level adolescent swimmers (Golby and Sheard, 2006). The study used the candidate gene association design, which involves searching for an association between a phenotype of interest and a known candidate gene. The gene might be chosen because of its genomic position or because it codes for the synthesis of a protein, which is hypothesized to contribute to the phenotype’s causal pathway (Balding, 2006). The 5-HTT gene was selected because the moderating effect of one of its polymorphism on the risk of developing maladaptive behavior patterns has become the major focus of studies of reactions to stress (e.g., Caspi et al., 2003). The sample was split into three groups on the basis of 5-HTT genotype: SS (individuals with two copies of the 5-HTT short allele), SL (individuals with one copy of the 5-HTT short allele and one copy of the 5-HTT long allele) and LL (individuals with two copies of the 5-HTT long allele). No significant associations were reported in this study. This was probably because the study was massively underpowered to detect an association between genetic variants and MT (Golby and Sheard, 2006).

The biggest problem for candidate gene association studies of complex traits is that their results are difficult to replicate; this is because the largest genetic effect sizes are much smaller than expected and the studies are underpowered to detect them (Plomin, 2013). Hundreds of thousands of participants are required for a genetic study to be adequately powered to reliably detect associations between common genetic variants and complex traits (Visscher, 2008).

## Discussion and Summary

The concept of MT has grown in popularity over the last decade and has broken out from its silo of sport psychology into other domains. In this paper, we systematically review empirical studies that explored the associations between MT, learning, educational and work performance, psychological well-being, personality, and other psychological attributes. The review indicates that MT is a vital trait across various contexts such as education, the workplace, and the military. Emotionally, mentally tough individuals are able to maintain greater levels of control and confidence under stressful situations, which might lead to better psychological well-being. Cognitively, they exhibit greater inhibition which allows them to commit to current tasks. Behaviorally, they are more likely to adopt problem-focused coping strategies to effectively manage stress, and make use of positive techniques such as motivational imagery and self-enhancing humor. These associations suggest that MT carries substantial implications for learning, academic results, work performance as well as a range of other achievement outcomes.

We have identified several limitations in the literature. Almost all MT studies employed self-report questionnaires to measure MT. Self-report measures are susceptible to socially desirable responding and biases in self-representation, thus may be discrepant from actual behaviors. Future research could consider combining self-report assessment of MT with assessment of MT from multiple raters (e.g., from teachers and parents when assessing students’ MT) to get a more reliable assessment of MT. An example of the use of independent assessment of mentally tough behaviors is the study by Hardy L. et al. (2014) who asked coaches to rate cricketers in their teams on MT. However, the extent to which independent assessments of mentally tough behaviors correlate with self-reported MT remains to be explored.

Some authors suggest that the existing models and measures of MT that were developed in sport contexts have limited predictive validity in task performance in non-athletic populations and in non-sport domains (e.g., Gucciardi et al., 2012; Hardy J.H. et al., 2014). However, we reviewed evidence that showed that MT is associated with cognitive, educational, and mental health outcomes in relatively heterogeneous non-athletic populations (e.g., Gerber et al., 2013a). Future research could attempt to develop measures and models of MT using non-athletic samples and to test the degree to which these new measures would predict performance across diverse settings.

Another limitation in the literature is the limited use of longitudinal designs to explore the directionality of the reported relationships between MT with other psychological traits. As such, it remains equivocal as to whether MT is a by-product of a host of positive cognitive attributes that are important for optimal performance; or whether being mentally tough is crucial for producing high levels of cognitive and behavioral performance. For example, it is possible that high cognitive and educational performance contributes to the development of MT, which in turns facilitates performance at school.

Behavioral genetics studies have suggested that, similar to other personality traits (e.g., Vukasović and Bratko, 2015), approximately half of the variation in MT can be accounted for by genetic factors. Despite that, molecular genetics research has not yet attempted to identify specific genes that contribute to variation in MT. Similarly, there is currently limited developmental research that has explored specific environmental factors that contribute to the remaining half of the variation (that can be accounted for by environmental factors) in MT from early childhood, throughout adolescence to adulthood. For this to happen, appropriate measures will need to be developed in order to assess MT across development, making sure that the measures are tapping into the same underlying construct at different ages. Longitudinal and developmental research over a long temporal span may provide significant insights as to which environmental factors can promote, sustain or attenuate the development of MT at different ages. Furthermore, there are currently no behavioral genetics studies that have explored the degree to which heritability estimates of MT change over time. It is possible that, similar to other psychological traits such as symptoms of stress and depression (Bergen et al., 2007), the heritability of MT increases with age. Such finding would suggest that environmental factors may have a more important role to play in MT early on in development. As such, training MT in childhood (rather that in adulthood) could be more effective in the field’s attempt to cultivate positive psychological capacities and to support achievement of desirable outcomes in education, mental health, and the workplace (Clough and Strycharczyk, 2012). However, there is, at present, a lack of empirical support for the importance of interventions to increase MT. Therefore, the suggestions regarding the necessity of developing MT interventions to boost performance remain speculative and we wanted to make this clear and separate it out from the solid research base described elsewhere in the paper.

Finally, a number of limitations in the literature create associated limitations in the present review. For example, considering the lack of replicated findings on the structure of MT (e.g., multidimensional vs. unidimensional), it remains challenging to identify specific similarities and differences between MT and, seemingly related constructs, such as hardiness, resilience, and grit. The studies reviewed here were highly heterogeneous in terms of the samples’ characteristics (e.g., typical students, athletes) and constructs assessed (e.g., various types of personality traits using different measures). As such, the current review is limited in making coherent general conclusions concerning the association between MT and the traits reviewed here. While all studies that have been reviewed here explored MT beyond the sport arena, many have used athlete samples. As such, future work should attempt to replicate the reported associations in representative samples derived from the general population.

Addressing some of the limitations of previous work can have significant theoretical and applied implications in the field of MT and personality research more generally. These implications involve identifying early on individuals who are at risk to exhibit low MT later in development; and intervening when it will be most beneficial for the individual to improve MT, a psychological trait that associates with optimal performance under pressure situations across diverse contexts.

## Author Contributions

JM has conducted the systematic review of the literature. YL and JM have equally contributed to the write up of this manuscript. PC has contributed to the write-up of this review. KP has contributed to the write-up of this review including specifying the focus of the review and the topic under investigation.

## Conflict of Interest Statement

The authors declare that the research was conducted in the absence of any commercial or financial relationships that could be construed as a potential conflict of interest.
