# Supplementary material for: Mental Toughness and Individual Differences in Learning, Educational and Work Performance, Psychological Well-being, and Personality: A Systematic Review
Source: Front Psychol. 2017 Aug 11;8:1345. doi: 10.3389/fpsyg.2017.01345 (PMC5554528; doi:10.3389/fpsyg.2017.01345)
Supplement: Supplementary file 1 [file Table_1.DOCX]

**Supplementary Material**

| Table S1 | | | | |
| --- | --- | --- | --- | --- |
| *Summary of Studies that Link Mental Toughness to Individual Differences in Cognition, Educational, Work and Military Performance, Psychological Wellbeing, Personality, Other Psychological Traits, and Genetics* | | | | |
| *Section 1: Cognition and Educational, Work, and Military Performance* | | | | |
| Arthur et al. (2015; Study 2) | 104 military recruits | MTMTI | Military training performance (final course grades) | MT predicted military training performance, *R*² = .31, *p* < .01 |
| Arthur et al. (2015; Study 3) | 134 military recruits | MTMTI | Military selection performance (pre-Para selection test) | MT predicted performance over and above that accounted for by physical fitness, ∆*R*² = .06, *p* < .01 |
| Crust et al. (2014) | 161 sport students | MTQ48 | Academic grades (end of year grades); academic progression (credits) | Positive correlations between MT and grades*,* *r* = .31, *p* < .01, and between MT and progression, *r* = .25, *p* < .01 |
| Dewhurst et al. (2012) | 60 students | MTQ48 | Recall performance (directed forgetting paradigm) | MT predicted directed forgetting, *R^2^* = .21, *p* < .01 |
| Delaney et al. (2015) | 120 undergraduates | MTQ18 | Big Five personality traits (Big Five Inventory); behavioural inhibition and activation (Behavioural Inhibition System and Behavioural Activation System Scales); recall performance (Directed forgetting paradigm) | Positive correlation between MT and conscientiousness, *r* = .41, *p* < .01, extraversion, *r* = .37, *p* < .01, agreeableness, *r* = .32, *p* < .01; positive correlation between MT and behavioural activation, *r* = .19, *p* < .01 and negative correlation between MT and behavioural inhibition, *r* = -.60, *p* < .01; MT predicted directed forgetting, *r* = .27, *p* < .05 |
| Godlewski et al. (2012) | 459 military recruits | MTQ18 | Normative and affective commitment (both developed from Meyer, Allen, and Smith (1993)); adjustment (The Organizational Socialization Inventory) | Positive correlation between MT and normative commitment, *r* = .11, *p* < .01 and affective commitment, *r* = .14, *p* < .01; positive correlation between MT and adjustment, *r* = .48, *p* < .01 |
| Gucciardi, Hanton, et al. (2015; Study 3) | 497 employees | MTI | Supervisor-rated work performance; perceived stress (Perceived Stress Scale) | MT predicted work performance directly, *ß* = .34, *p* < .001 and indirectly through perceived distress, standardized indirect effect estimate = .24, 95% CI [.17, .31] *p* < .001 |
| Gucciardi, Hanton, et al. (2015; Study 4) | 203 undergraduates | MTI | Thriving (Thriving Scale); positive emotions (Tamir et al., 2007) and negative emotions (Depression Anxiety Stress Scales); academic and social goal progress | Both within-person and between-person differences in MT predicted negative emotional states (R^2^_within_ = .1, R^2^_between_ = .21), positive emotions (R^2^_within_ = .33, R^2^_between_ = .75), thriving (R^2^_within_ = .41; R^2^_between_ = .87), and academic (R^2^_within_ = .15, R^2^_between_ = .53) and social goal progress (R^2^_within_ = .03, R^2^_between_ = .45) |
| Gucciardi, Hanton, et al. (2015; Study 5) | 115 military recruits | MTI | Military selection test outcome | MT predicted success in selection test, *b* = 1.25, *p* < .05 |
| J.H. Hardy et al. (2014) | 120 university students | MTQ18; domain-specific measure of MT | Complex task learning (Unreal Tournament 2004) | Non-significant relationships between MT and any learning criteria; domain-specific MT predicted post-practice self-efficacy, *R^2^* = .07, *p* < .05, and performance, *R^2^* = .06, *p* < .05 |
| Marchant et al. (2009) | 522 participants working in the UK | MTQ48 | Managerial position | Significant effect of management on MT, *F* (3, 518) =14.64, *p* < .001 |
| St Clair-Thompson et al. (2015; Study 1) | 159 students | MTQ48 | Academic attainment (latest national curriculum levels); academic attendance (percentage of attendance in previous full academic term) | Positive correlations between MT and academic attainment, *r* = .22, *p* < .01, and academic attendance, *r* = .22, *p* < .01 |
| St Clair-Thompson et al. (2015; Study 2) | 295 adolescents | MTQ48 | Oppositional behaviour, cognitive problems/inattention, hyperactivity and ADHD (Conners’ Teachers Rating Scale Revised) | Negative correlations between MT and oppositional behaviour, *r* = -.23, *p* < .01, cognitive problems/inattention, *r* = -.17, *p* < .01, hyperactivity, *r* = -.14, *p* < .01, and ADHD, *r* < -.15, *p* < .01 |
| St Clair-Thompson et al. (2015; Study 3) | 93 students | MTQ48 | Peer relationships (Social Inclusion Survey; social acceptance scale of Self-Perception Profile) | Positive correlations between MT and social inclusion, *r* = .22 to .24, *p* < .01 and social acceptance, *r* = .38, *p* < .01 |
| St Clair-Thompson et al. (2016; Study 1) | 105 pupils | MTQ48 | Self-esteem (Rosenberg’s Self-Esteem Scale); concerns about school (School Concerns Questionnaire) | Positive correlations between MT and self-esteem, *r* = .23 to .83, *p* < .01 and negative correlations between MT and school concerns, *r* = -.27 to -.52, *p* < .01 |
| St Clair-Thompson et al. (2016; Study 2) | 200 undergraduate students | MTQ48 | College adjustment (Student Adaptation to College Questionnaire) | Positive correlations between MT and college adjustment, *r* = .19 - .70, *p* < .01 |
| *Section 2: Psychological Wellbeing* | | | | |
| Brand et al. (2014a) | 92 adolescents | MTQ48 | Sleep disturbance (Insomnia Severity Index); daytime sleepiness (Epworth  Sleepiness Scale); objective sleep quality (EEG monitoring) | Positive correlation between MT and sleep efficiency, *r* = .74, *p* < .001; MT was associated with fewer awakenings after sleep onset, *r* = -.69, *p* < .001; MT associated with lower daytime sleepiness, *r* = -.88, *p* < .001, and fewer sleep complaints, *r* = .73, *p* < .001; sleep EEG indicative of better objective sleep quality |
| Brand et al. (2014b) | 284 adolescents | MTQ48 | Perceived stress (Perceived Stress Scale); depressive symptoms (Beck Depression Inventory); sleep disturbance (Insomnia Severity Index); daytime sleepiness (Epworth  Sleepiness Scale) | Positive correlation between MT and sleep quality, *r* = .37, *p* < .001, sleep duration, *r* = .21, *p* < .001; negative correlation between MT and sleep onset latency, *r* = -.19, *p* < .001, awakenings after sleep onset, *r* = -.37, *p* < 001, depressive symptoms, *r* = -.65, *p* < .001, and perceived stress, *r* = -.73, *p* < .001 |
| Brand, Hatzinger, et al. (2015) | 37 adolescents | MTQ18 | Sleep disturbance (Insomnia Severity Index) | Normal and good sleep at the age of five demonstrated more favourable scores of MT, at the age of 14, relative to poor sleepers, *F*(2, 36) = 8.52, *p* < .001 |
| Brand, Kalak, Gerber, Clough, Lemola, Pühse, et al. (2016) | 1475 adolescents | MTQ48 | Quality of life (KIDSCREEN-  52); physical activity (adaption of the International Physical Activity Questionnaire); sleep disturbance (Insomnia Severity  Index) | Positive correlation between MT and quality of life, *r* = .17 to .51, *p* < .001 and negative correlation between MT and sleep disturbance, *r* = -.25, *p* < .001; no association between MT and physical activity |
| Brand, Kalak, Gerber, Clough, Lemola, Sadeghi Bahmani, et al. (2016) | 1361 adolescents | MTQ18 | Physical activity (adaption of the International Physical Activity Questionnaire) | *F*(2, 1355) = 6.50, *p* < .01 |
| Cowden et al. (2014) | 16 elite tennis players | SMTQ | Learned resourcefulness (Self-Control Schedule); trait anxiety (Sports Competition Anxiety Test) | A positive correlation between MT and learned resourcefulness, *r* = .79, *p* < .001, but no association with competitive trait anxiety |
| Cowden et al. (2016) | 351 tennis players | SMTQ | Resilience (Resilience Scale for Adults); stress (modified version of the Recovery-Stress Questionnaire for Athletes) | Positive correlation between MT and resilience, *r* = .59, *p* < .001 (one-tailed) and negative correlation with stress, *r* = -.44, *p* < .001 (one-tailed) |
| Crust (2009) | 112 university athletes | MTQ48 | Affect intensity (Affect Intensity Measure) | Non-significant correlation between affect intensity and MT |
| Gerber, Brand, et al. (2013) | 865 students | MTQ18 | Stress (German version Adolescent Stress Questionnaire), depressive symptoms (German version CES-D), life satisfaction (Satisfaction with Life Scale) | A negative correlation between MT and stress, *r* = -.42, *p* < .001; MT was predictive of depressive symptoms at 10-month follow-up, *ß* = -.18, *p* < .001; MT was predictive of life satisfaction at 10-month follow-up, *ß* = .12, *p* < .001 |
| Gerber et al. (2012) | 284 high school students | MTQ48 | Moderate-to-vigorous physical activity (International Physical Activity Questionnaire) | Significant effect of moderate physical activity on MT, Wilk’s *Λ* = .84 |
| Gerber, Kalak, et al. (2013) | Sample 1: 284 high school students  Sample 2: 140 undergraduates | MTQ48 | Perceived stress (Perceived Stress Scale), depressive symptoms (Beck Depression Inventory) | Negative correlations between MT and stress, *r* = -.65, *p* < .001, and depressive symptoms, *r* = -.65, *p* < .001 in both samples |
| Gerber, Feldmeth, et al. (2015) | 54 vocational students | MTQ18 | Perceived stress (Adolescent  Stress Questionnaire); burnout (Shirom-Melamed Burnout Measure) | Negative correlation between MT and stress, *r* = -.49, *p* < .001 and burnout, *r* = -.28, *p* < .05 to *r* = -.43, *p* < .01 |
| Gerber, Lang, et al. (2015) | 56 vocational students | MTQ18 | Burnout (Shirom Melamed Burnout Measure); moderate-to-vigorous physical activity (International Physical Activity Questionnaire) | Students who accomplished moderate-to-vigorous physical activity reported higher MT, *R^2^* = .23, *p* < .01, and had fewer burnout symptoms |
| Gucciardi and Jones (2012) | 226 cricketers | CMTI | Psychological distress (Depression, Anxiety, and Stress Scale-21) | Negative correlation between MT and stress, *r* = -.14, *p* < .05 to *r* = -.30, *p* < 01, anxiety, *r* = -.17 to -.25, *p* < .01, and depression, *r* = -.10 to -.24, *p* < .01 |
| Hannan et al. (2015) | 117 community participants and undergraduate students | MTI | Physical activity (International Physical Activity Questionnaire); intentions to engage in physical activity (Theory of Planned Behavior Questionnaire) | Significant association between intentions and physical activity at moderate and strong levels of MT, *β* = .28, *p* = .001, and *β* = .40, *p* = .011, respectively |
| Jin and Wang (2016) | 217 international students | SMTQ | Adult attachment (Experiences in Close Relationship Scale); life-satisfaction (Satisfactory with Life Scale); psychological distress (Depression, Anxiety, and Stress Scale-21) | Negative correlation between MT and attachment avoidance, *r* = -.23, *p* < .01 and attachment anxiety, *r* = -.32, *p* < .01; positive correlation between MT and life satisfaction, *r* = .32, *p* < .01; negative correlation between MT and stress, *r* = -.36, *p* < .01, MT and anxiety, *r* = -.38, *p* < .01, and MT and depression, *r* = -.41, *p* < .01 |
| Kaiseler et al. (2009) | 482 athletes | MTQ48 | Stressor appraisal; coping and coping effectiveness (MCOPE) | Overall MT predicted both lowered stress intensity, *R^2^* = .03, *p* < .001, and greater perceptions of control, *R^2^* = .04, *p* < .001 |
| L. Hardy et al. (2014) | 214 male cricketers | Informant-rated MTI | Reward and punishment sensitivity (Corr’s (2001) transformations of the short version of the Eysenck Personality Questionnaire-Revised) | When reward sensitivity was low, there was a positive correlation between MT and punishment sensitivity, *t(211)* = 4.96, *p* < .01; when reward sensitivity was high, there was a negative correlation between MT and punishment sensitivity, *t(211)* = –6.27, *p* < .01 |
| Mutz et al. (in press) | 364 participants of diverse backgrounds | MTQ48 | Depressive symptoms (Clinically Useful Depression Outcome Scale; Patient Health Questionnaire 9), cognitive reappraisal and expressive suppression (Emotion Regulation Questionnaire) | Negative correlations between MT and both measures of depressive symptoms (CUDOS *r* = -.53, *p* < .001 and PHQ-9 *r* = -.49, *p* < .001) and with expressive suppression, *r* = -.19, *p* < .001; positive correlation between MT and cognitive reappraisal, *r* = .26, *p* < .001; the relationship between MT and depressive symptoms was mediated by individual differences in expressive suppression |
| Nicholls et al. (2011) | 206 athletes | MTQ48 | Coping self-efficacy (Coping Self-Efficacy Scale); coping effectiveness (CE scale) | Positive relationship between MT and coping self-efficacy, *r* = .35, *p* < .01 and coping effectiveness, *r* = .23, *p* < .01 |
| Sabouri et al. (2016) | 341 adults | MTQ18 | Physical activity (short version of International Physical Activity Questionnaire) | Positive relationship between MT and vigorous physical activity, *r* = .19, *p* < .01 |
| Sadeghi Bahmani et al. (2016) | 77 adolescents | MTQ18 | Sleep disturbance (Insomnia Severity Index); internalizing and externalizing problems, hyperactivity, negative peer relationships and prosocial behaviour (Strengths and Difficulties Questionnaire) | Negative correlation between MT and sleep disturbances, *r* = -.45, *p* < .05; fewer internalising and externalising problems, more positive peer relationships, and more prosocial behaviour at age five predicted MT at age 14, *R*² = .16, *p* < .001 |
| Stamp et al. (2015) | 168 undergraduates | MTQ48 | Psychological wellbeing (Scales of Psychological Wellbeing) | Positive correlations between all components of MT and all subscales of psychological wellbeing, *r*’s ranged from .17 to .77, *p* < .05 |
| *Section 3: Personality and Other Psychological Traits* | | | | |
| Brand, Kirov, et al. (2015) | 346 undergraduates | MTQ48 | Perfectionism (the Frost Multidimensional Perfectionism Scales) | Negative correlations between MT and concerns over mistakes, *r* = -.16, *p* < .01, and personal standards, *r* = -.28, *p* < .001 |
| Crust & Azadi (2010) | 67 male athletes | MTQ48 | Psychological strategies (Test of Performance Strategies) | Positive correlations between MT and emotional control, *r* = .30, *p* < .01, self-talk, *r* = .37, *p* < .01, and relaxation strategies, *r* = .26, *p* < .01 |
| Crust and Keegan (2010) | 105 university athletes | MTQ48 | Attitudes to risk-taking (Attitudes Towards Risks Questionnaire) | A positive correlation between overall MT and attitudes towards physical risk-taking, *r* = 0.3, *p* < .01, but not psychological risk-taking |
| Crust and Swann (2013) | 135 club and university athletes | MTQ48 | Dispositional flow (Dispositional Flow Scale-2) | A positive correlation between overall MT and global flow experiences, *r* = .65, *p* < .001 |
| Gucciardi (2010) | 214 male Australian footballers | Australian Football Mental Toughness Inventory | Achievement goals (Achievement Goals Questionnaires-Sport); sport motivation (Sport Motivation Scale-6) | Positive correlations between MT subscales and mastery-approach, *r* = .46 to .60, and performance-approach goals, *r* = .11 to .21, intrinsic motivation, *r* = .35 to .47, identified regulation, *r* = .25 to .29, and external regulation, *r* = .23 to .28; all *p* < .05 |
| Gucciardi and Jones (2012) | 226 community cricketers | CMTI | Developmental assets (Developmental Assets Profile | MT positively predicted developmental assets possession, $\eta$^2^ = .13, *p* < .001 |
| Gucciardi, Jackson, et al. (2015) | 347 adolescent tennis players | Informant-rated measure | Fear of failure; inspiration (Inspiration Scale); passion (Passion Scale) | Positive correlations between mentally tough behaviours and harmonious passion, $\beta$ = .26, *p* < .01, and frequency of inspiration,$\beta$ = .32, *p* < .001; negative correlations between mentally tough behaviours and fear of failure, $\beta$ = -.32, *p* < .001, and obsessive passion $\beta$ = -.15, *p* < .01 |
| Madrigal et al. (2013) | 143 college basketball athletes | MTS | Self-efficacy (Self-Efficacy Scale); self-esteem (Rosenberg Self-Esteem Scale); flow (Flow State Scale); social commitment (Social Responsibility Scale) | Positive correlations between MT and flow, *r* = .49, *p* < .05, self-efficacy, *r* = .22, *p* < .05, and self-esteem, *r* = .24, *p* < .05; no correlation between MT and social commitment |
| Mahoney et al. (2014) | 221 cross-country runners | MTI | Basic needs satisfaction (Basic Needs Satisfaction in Sport Scale); thwarting (Psychological Needs Thwarting Scale); psychological health (Mental Health Continuum Short Form) | Positive correlations between MT and psychological needs satisfaction, *r* = .59, *p* <.01, and between MT and positive affect, *r* = .40, *p* < .01; negative correlations between MT and psychological needs thwarting, *r* = -.38, *p* < .01, and between MT and negative affect, *r* = -.37, *p* < .01 |
| Mattie and Munroe-Chandler (2012) | 151 college athletes | MTQ48 | Imagery use (The Sport Imagery Questionnaire) | Motivational imagery predicted all subscales of MT, *r*’s ranged from .25 to .59, *p* < .01 |
| Meggs et al. (2014) | 105 athletes | SMTQ | Self-concept organisation (self-descriptive attribution task) | Negative self-content predicted lower levels of MT, *β* = −.31, *p* = .003; positive-integrated self-organisations predicted higher levels of MT, *β* = −.64, *p* < .001; positively compartmentalized self-concepts predicted higher levels of overall MT, *β* = .18, *p* = .12 |
| Nicholls et al. (2008) | 667 athletes | MTQ48 | Optimism and pessimism (Life Orientation Test); coping (Coping Inventory for Competitive Sport) | Positive correlation between MT and optimism, *r* = .56, *p* < .01; negative correlation between MT and pessimism, *r* = -.46, *p* < .01; MT was associated with more problem or approach coping strategies and less avoidance coping strategies |
| Nicholls et al. (2015; Study 1) | 531 athletes | MTQ48 | Emotional intelligence (The Trait Emotional Intelligence Questionnaire); resilience (Connor-Division Resilience Scale) | MT positively predicted resilience, *β* = .80, *p* < .01, and was positively predicted by emotional intelligence, γ = .92, *p* < .01 |
| Nicholls et al. (2015; Study 2) | 522 athletes | MTQ48 | Self-efficacy (The General Self-Efficacy Scale); sport motivation (The Sport Motivation Scale) | MT positively predicted self-efficacy, *β*  = .60, *p* < .01, and was negatively predicted by introjected regulation, γ  = -.25, *p* < .01, and amotivation, γ  = -.35, *p* < .01 |
| Sabouri et al. (2016) | 341 adults | MTQ18 | Machiavellianism (MACH-$I$V); narcissism (Narcissistic Personality Inventory); psychopathy (Psychopathy Scale-$III$) | Positive correlations between MT and Machiavellianism, *r* = .45, *p* < .01, narcissism, *r* = .50, *p* < .01, and psychopathy *r* = .20, *p* < .01 |
| Schaffer et al. (2016) | 173 golfers | MTI | Competition anxiety (Sport Competition Anxiety Test); motivation (Sport Motivation Scale-$II$) | Positive correlations between MT and autonomous forms of motivation, *r* = .39 to .57, *p* < .001; negative correlations between MT and amotivation, *r* = -.23, *p* = .001, and competition anxiety, *r* = -.19, *p* = .02 |
| *Section 4: Genetics*  rp = observed phenotypic correlation rg = genetic correlation re = non-shared environmental correlations | | | | |
| Horsburgh et al. (2009) | 219 pairs of adult twins | MTQ48 | Big five personality traits (240-item NEO-PI-R) | Heritability estimate for overall MT was .52, with estimates for MT components ranging from .36 to .56; phenotypic correlations between total MT and all MT subscales (except “control over life”) with the Big 5 personality traits could be fully accounted for by additive genetic and non-shared environments:  MT-Neuroticism: rp = -.64, rg = -.90  MT-Extraversion: rp = .45, rg = .53  MT-Openness: rp = .18, rg = .26  MT-Agreeableness: rp =.20, rg = .38  MT-conscientiousness: rp =.43, rg = .39 |
| Onley et al. (2013) | 210 pairs of same-sex twins | MTQ48 | Dark triad traits (Narcissistic Personality Inventory, Self-report Psychopathy Scale-III, MACH-IV) | Correlations between narcissism and MT factors were largely influenced by non-shared environment except challenge, which was best explained by genetic factors (rp ranged from .13 to .23); correlations between psychopathy and commitment, control and confidence were fully accounted for by common genetic factors rp ranged from -.18 to -.23); Machiavellianism and all MT factors were fully explained by genetic and non-shared environmental factors (rp ranged from .24 to .30) |
| Veselka et al. (2009) | 201 pairs of adult twins | MTQ48 | Humour styles (Humour Styles Questionnaire) | Phenotypic correlations between MT and humour styles were primarily attributable to genetic and non-shared environmental factors: MT-affiliative humour style: rp = 26, rg = .20, re = .31; MT-self-enhancing humour style: rp = 40, rg = .63, re = .23; MT-self-defeating humour style: rp = -.15, rg = -.31, re = -.02 |
| Veselka et al. (2010) | 219 pairs of same-sex twins | MTQ48 | Big five personality traits (NEO-PI) | A general factor of personality (GFP) was extracted, representing high MT, extraversion and conscientiousness, and low neuroticism; Genetic (53%) and nonshared environmental factors (47%) fully accounted for variance in GFP |
| Golby and Sheard (2006**)** | 31 high-achieving adolescent swimmers | The Psychological Performance Inventory | 5-HTT genotype (DNA extraction) | No significant association between MT and 5-HTT genotype |
